# Spinal Anesthesia Results in Lower Costs Compared to General Anesthesia for Patients Undergoing Lumbar Fusion—A Matched Cohort Study

**DOI:** 10.3390/jcm14113851

**Published:** 2025-05-30

**Authors:** Favour C. Ononogbu-Uche, Abdullah Wael Saleh, Felix Toussaint, Taylor Wallace, Joshua Woo, Matthew T. Morris, Christopher I. Shaffrey, William M. Bullock, Nicole R. Guinn, Muhammad M. Abd-El-Barr

**Affiliations:** 1 Department of Neurosurgery, Duke University, Durham, NC 27710, USA; abdullah.saleh@duke.edu (A.W.S.); felixtoussaint@students.aucmed.edu (F.T.); t.wallace@duke.edu (T.W.); joshua.woo@duke.edu (J.W.); christopher.shaffrey@duke.edu (C.I.S.); 2 Duke School of Medicine, Duke University, Durham, NC 27710, USA; matthew.morris@duke.edu; 3 Department of Orthopedic Surgery, Duke University, Durham, NC 27710, USA; 4 Department of Anesthesiology, Duke University, Durham, NC 27710, USA; william.bullock@duke.edu (W.M.B.); nicole.guinn@duke.edu (N.R.G.)

**Keywords:** TLIF, lumbar surgery, cost-effectiveness, spinal anesthesia, general anesthesia, spine surgery

## Abstract

**Background/Objectives**: Degenerative lumbar spine disease (DLSD) is increasingly managed with minimally invasive surgery (MIS) and evolving anesthesia methods. While general anesthesia (GA) remains standard, spinal anesthesia (SA) may offer faster recovery and fewer side effects. This study compares the clinical and economic outcomes of GA versus SA in transforaminal lumbar interbody fusion (TLIF). **Methods**: A retrospective review of 18 TLIF patients (2018–2022) was performed, with 9 patients in each cohort. Patients were matched by demographics and procedure type. Data collected included operative time, blood loss, complications, postoperative opioid utilization, and 30-day readmissions. Costs were analyzed in categories: anesthesia, implants, inpatient care, operating room (OR) supplies, OR time, and PACU fees, using Wilcoxon Rank T-tests and Pearson Chi-Squared tests. **Results**: Clinical outcomes such as blood loss, and operative time were similar between groups. However, SA patients had significantly shorter LOS compared to GA (SA: 12 h vs. GA: 84 h, % difference: −150%, *p* = 0.04). Additionally, SA patients had lower total direct costs ($27,881.85 vs. $35,669.01; *p* = 0.027). Significant cost reductions with SA were noted in OR supplies/medications ($7367.93 vs. $10,879.46; *p* = 0.039) and inpatient costs ($621.65 vs. $3092.66; *p* = 0.027). Within these categories, reductions were observed for intravenous solutions, sedatives/anesthetics, pressure management, labs, imaging, evaluations, hospital care, and medications. Although costs for implants, anesthesia care, OR time, and PACU fees were lower with SA, these differences did not reach statistical significance. **Conclusions**: In TLIF for DLSD, SA provides significant economic advantages over GA while yielding comparable clinical outcomes. These results support SA as a cost-effective alternative, warranting further prospective studies to confirm these findings.

## 1. Introduction

Degenerative lumbar spine disease (DLSD), including conditions such as spondylolisthesis, symptomatic disk degeneration, and spinal stenosis, has become increasingly prevalent, leading to a rising demand for effective management strategies [1]. With advancements in minimally invasive spine surgery (MIS) techniques, a growing proportion of patients with DLSD are seeking surgical interventions, with laminectomy, discectomy, and fusion among the most performed procedures [2]. Compared to traditional open surgery, MIS techniques, such as the minimally invasive Transforaminal Lumbar Interbody Fusion (MIS-TLIF), yield comparable clinical outcomes while reducing perioperative complications, blood loss, and hospital stays, marking a paradigm shift in spine care [3]. This shift is complemented by advancements in anesthesia, enhancing analgesic efficacy, reducing risks, and accelerating recovery.

Traditionally, lumbar spine surgeries have relied on general anesthesia (GA), which relies on reversible unconsciousness, analgesia, and suppression of autonomic reflexes to provide optimal surgical conditions [4]. GA is widely applied across spine surgery and continues to be the most prevalent anesthetic technique implemented in spine surgery [5]; however, it is contraindicated in certain patients, such as those who cannot tolerate prone positioning [6]. GA may cause postoperative nausea and vomiting, and carries a risk of serious complications, such as cardiovascular instability, especially in elderly patients [1,7]. Recently, spinal anesthesia (SA) has emerged as a promising alternative for spine surgery [8]. Administered intrathecally, SA achieves regional anesthesia without requiring intubation, allowing patients to remain conscious while pain-free [9]. SA protects hemodynamic stability, enables real-time neurological monitoring, and often results in a smoother recovery [10,11]. Additionally, SA has been shown to be associated with improved post-operative patient outcomes such as reduced length of stay, opioid use, and time to ambulation [12,13].

While the pharmacologic, physiologic, and peri-operative effects of GA and SA have been explored, the economic implications of each anesthesia type remain less understood. Though previous studies have examined cost differences between SA and GA [14,15,16,17,18,19,20,21,22,23,24,25,26], only two have been specific to the TLIF [16,17]. Furthermore, none have done so while using a matched cohort to ensure precise comparisons. Given that cost considerations strongly impact decision-making for both patients and healthcare systems [27], evaluating cost-effectiveness across anesthetic techniques is essential—particularly as MIS techniques continue to evolve [28,29]. This study addresses this gap through a retrospective, carefully matched cohort analysis, comparing both clinical outcomes and costs associated with awake spine surgery using SA versus traditional GA. By controlling for key variables across patient groups, this analysis aims to offer an accurate cost comparison across methods, providing insights that could promote cost-effective practices and inform anesthesia selection in lumbar surgery, particularly TLIFs. This approach has the potential to enhance clinical decision-making by quantifying both economic and clinical impacts, thereby advancing value-based care in spinal procedures.

## 2. Materials and Methods

### 2.1. Study Design

This retrospective analysis reviewed electronic medical records of 18 patients who underwent transforaminal lumbar interbody fusion (TLIF) between January 2018 and December 2022. Outcome data were collected from these records, while cost data were obtained from the hospital’s financial department. To ensure confidentiality, all patient identifiers were removed from the cost data. The study compared the cost-effectiveness and clinical outcomes of different anesthetic modalities.

### 2.2. Cohort Development and Data Collection

The study included a single-surgeon, single-center retrospective review of a prospectively maintained database. Patients were divided into two cohorts: those who received spinal anesthesia (SA) and those who underwent general anesthesia (GA), matched for demographics including age, body mass index (BMI), spinal level, surgical technique (percutaneous vs. minimally invasive), procedure type, and relevant comorbidities including cardiac disease, pulmonary disease, renal disease, hypertension, and diabetes. Matching was performed using coarsened exact matching (CEM). The decision between SA and GA was based on patient preference following detailed discussions of both anesthetic options, and patients with contraindications to awake surgery, such as difficult airways, were excluded from the SA cohort. No exclusion criteria were applied for age or BMI. Both cohorts followed similar preoperative counseling and had identical admission and discharge protocols. All patient data were de-identified and stored in accordance with IRB regulations. Clinical outcomes, including estimated blood loss (EBL), intraoperative complications, 30-day readmission rates, operative time, and length of stay (LOS) were collected from electronic medical records. Additionally, we collected postoperative opioid consumption in oral morphine equivalents (OME) for the first 6 h post operation, to enable us to collect data at a time frame applicable for all patients. We defined intraoperative complications as adverse events occurring during surgery requiring immediate intervention or potential to impact patient safety, such as screw revision due to CSF leakage, vertebral artery injury, premature termination of the procedure, nerve root damage, and cement leakage. Patient characteristics were obtained from hospital intake forms and preoperative surgical notes, while intraoperative and postoperative complications were documented from operative notes and PACU nursing records.

### 2.3. Cost Data Collection

Cost data were obtained from the hospital’s revenue and finance team, who were blinded to the study outcomes. All costs were listed as net hospital costs (the amount the hospital paid for goods and services), excluding indirect costs such as administration, cafeteria, and laundry services. The analysis included only direct, non-fixed costs attributable to patient care. Surgeon fees and implantable device costs were excluded as they were not expected to differ based on anesthetic modality.

Costs were categorized into the following major cost groups, each with their own minor cost categories (Table 1):

1. Anesthesia care costs (including anesthesiologist fees)
2. Implant costs (rods, screws, cages, grafts, etc.)
3. Inpatient costs (recovery support staff, medical supplies, drugs, etc.)
4. OR supplies/medications (staff, medical supplies, sterilization, drugs, etc.)
5. OR time fees
6. PACU time fees

Cost calculations were based on activity code descriptions, units of service/goods used, and the corresponding unit costs. For example, the cost for two units of “MATRIX FLOSEAL NDLS HUMAN THROMBIN (10 mL)” at $229.40 per unit amounted to $458.80. Each item was categorized into one of the six cost categories and further categorized into its associated sub-category.

### 2.4. Anesthetic Technique

For spinal anesthesia, the patient was seated with the spine flexed and legs hanging. The appropriate spinal level, typically L3–L4, was identified by palpation. Following sterilization and draping, a midline approach was used to access the intrathecal space. A 25-gauge, 90 mm Quincke spinal needle was inserted and advanced cephalad, passing through the skin, subcutaneous fat, supraspinous and interspinous ligaments, and the ligamentum flavum. Upon entering the dura and subarachnoid space and observing cerebrospinal fluid (CSF), 10–15 mg of 0.5% isobaric bupivacaine was injected to induce anesthesia. All patients received intravenous propofol and opioid sedation intraoperatively to maintain comfort throughout the procedure.

### 2.5. Cohort Development Variables and Outcome Measurements

Demographic data, including age, sex, BMI, medical comorbidities, and American Society of Anesthesiologists Physical Status (ASA-PS), were collected. Operative variables recorded included anesthetic modality, procedure type, spinal levels involved, fluoroscopy time, LOS, 30-day readmission rates, EBL, six-hour postoperative opioid utilization, and disposition. Intraoperative complications and operative times were measured, including total time spent in the operating room (OR), anesthesia duration, and surgical time (from incision to closure).

### 2.6. Statistical Analysis

Patients in the SA cohort were compared to demographically matched patients in the GA cohort. Mann–Whitney U test was used to compare means of continuous variables without normal distribution, and the Pearson Chi-Squared Test for independence for categorical variables. The analysis included total hospital costs, operative time, LOS, EBL, postoperative opioid utilization, intraoperative complications, and 30-day readmission rates.

## 3. Results

### 3.1. Demograohics and Non-Cost Outcomes

The study included 18 patients who underwent lumbar spinal fusion for degenerative spinal disease (DSD), with 9 patients in the spinal anesthesia (SA) group and 9 patients in the general anesthesia (GA) group. Both groups were successfully matched based on patient and procedural characteristics.

The SA cohort had a mean age of 65.8 ± 6.8 (*p* = 0.37), BMI of 27.7 ± 3.3 (*p* = 0.36), and a median ASA score of 2 (*p* = 0.11), respectively. The GA cohort had a mean age of 67.1 ± 7.9 years (*p* = 0.37), BMI of 29.6 ± 5.1 (*p* = 0.36), and median ASA score of 3 (*p* = 0.11) (Table 2). Both cohorts had comparable comorbidities, including smoking history, hypertension, diabetes, heart disease, pulmonary disease, renal disease, and prior spine surgery. LOS was significantly different between the SA and the GA groups with SA having a shorter median LOS (SA: 12 h vs. GA: 84 h, % difference: −150%, *p* = 0.04). All other non-cost clinical outcomes, such as estimated blood loss (EBL), operative time (OR time), postoperative opioid utilization, disposition, and patient-reported outcome measures (PROMs), showed no significant variability between groups. No intraoperative complications were observed in either group. PROMIS scores assessed included the Oswestry Disability Index (ODI), Visual Analog Scale for Back Pain (VAS-B), Visual Analog Scale for Leg Pain (VAS-L), PROMIS Physical Function T-Score, and PROMIS Pain Interference T-Score. Full demographic and outcome data are provided in Table 2 and Table 3, respectively.

### 3.2. Comparative Cost Analysis

#### 3.2.1. Total Cost Differences

**Figure 1:**
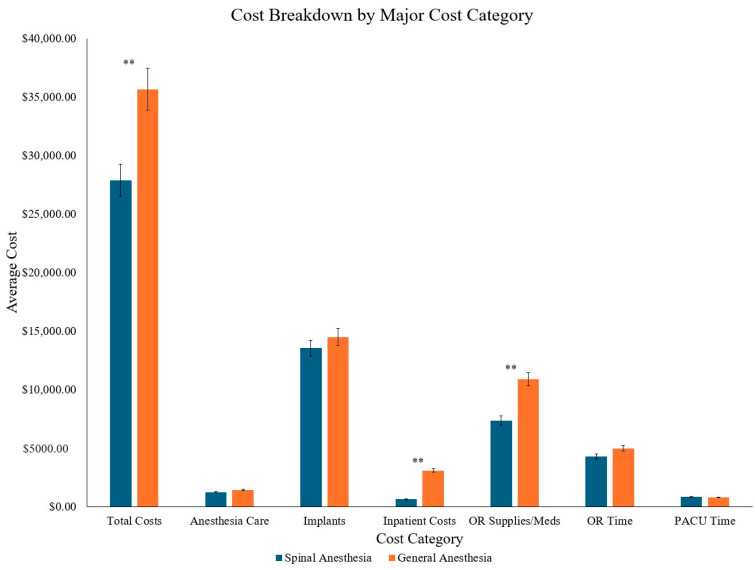
Cost differences across all major categories. ** represent significance (*p* < 0.05).

The total direct cost of transforaminal lumbar interbody fusion (TLIF) surgery was significantly lower in the SA group compared to the GA group (SA: $27,881.85 vs. GA: $35,669.01; % difference: −24.51%, *p* = 0.027). Each of the six primary cost categories showed lower costs in the SA group (Figure 1).

#### 3.2.2. Operating Room (OR) Supplies/Medications

**Figure 2:**
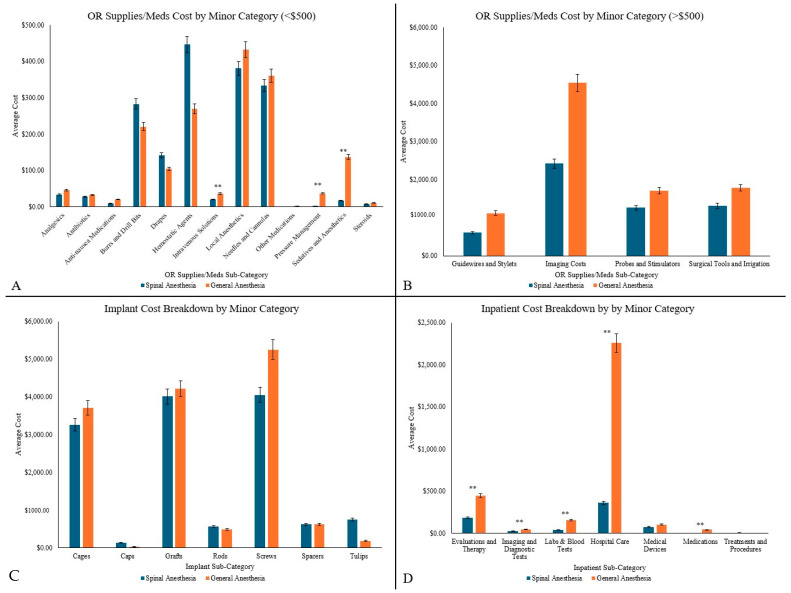
Cost differences across three major cost categories and their minor cost categories such as (**A**) OR Supplies/Medications costs less than $500, (**B**) OR Supplies/Medications costs greater than $500, (**C**) Implant costs, and (**D**) Inpatient costs. ** represent significance (*p* < 0.05).

OR supplies/med costs were significantly lower in the SA group (SA: $7367.93 vs. GA: $10,879.46; % difference: −38.49%, *p* = 0.039). Sub-analysis showed these cost differences to be driven by Intravenous Solutions (SA: $21.04 vs. GA: $36.67, % difference: −54.17%, *p* = 0.027), Sedatives and Anesthetics (SA: $17.53 vs. GA: $137.13, % difference: −154.66%, *p* = 0.004), and Pressure Management (SA: $2.70 vs. GA: $37.07, % difference: −172.84%, *p* = 0.02) (Figure 2A,B).

#### 3.2.3. In-Patient Costs

Inpatient costs were also significantly lower in the awake patients (SA: $621.65 vs. GA: $3092.66, % difference: −133.05%, *p* = 0.027). Within this category, sub-analysis identified further significant differences in Labs and Blood Tests (SA: $38.75 vs. GA: $155.93, % difference: −120.38%, *p* = 0.012), Imaging and Diagnostic Tests (SA: $28.90 vs. GA: $49.02, % difference: −51.64%, *p* = 0.027), Evaluations and Therapy (SA: $185.40 vs. GA: $446.02, % difference: −82.55%, *p* = 0.027), Hospital Care (SA: $360.52 vs. GA: $2257.79, % difference: −144.92%, *p* = 0.042), and Medications (SA: $2.40 vs. GA: $43.30, % difference: −178.99%, *p* = 0.012) (Figure 2C,D).

#### 3.2.4. Implant Costs

Implant costs were lower in the SA group than in the GA group, though this difference was not statistically significant (SA: $13,542.47 vs. GA: $14,502.91; % difference: −6.84%, *p* = 0.203), with no significant differences observed in subcategories.

#### 3.2.5. Anesthesia Care Costs

The SA group showed lower anesthesia care costs compared to the GA group, though this was not statistically significant (SA: $1246.52 vs. GA: $1422.37, % difference: 13.18%, *p* = 0.098).

#### 3.2.6. OR Time Costs

The cost for OR time was lower in the SA group but did not reach statistical significance (SA: $4267.14 vs. GA: $5062.32, mean difference: 17.05%, *p* = 0.113).

#### 3.2.7. Post-Anesthesia Care Unit (PACU) Time

PACU time costs were lower in the SA group (SA: $796.15 vs. GA: $819.33, mean difference: −2.87%, *p* = 0.822), with no statistically significant difference between groups.

## 4. Discussion

### 4.1. Summary of Key Findings

This study indicates that spinal anesthesia (SA) provides a clinically viable, cost-effective alternative to general anesthesia (GA) in transforaminal lumbar interbody fusion (TLIF) surgery. SA showed significantly reduced intraoperative and postoperative costs compared to GA. Overall, the SA group showed consistent cost savings across all major and minor cost categories, with notable significance in total overall costs, OR supplies/medications (sedative and anesthetic, intravenous solutions, and blood pressure management, respectively) and inpatient (labs and blood tests, imaging and diagnostics, evaluations and therapies, hospital care and medications, respectively) costs groups, respectively. The cost savings in SA patients were observed without compromising patient outcomes, underscoring its suitability for select patient populations. With comparable clinical metrics across both groups, SA offers a promising approach to streamline resource utilization while preserving surgical efficacy. These findings support a strategic role for SA in TLIF, especially as healthcare systems prioritize cost-containment alongside high standards of care.

### 4.2. Interpretation of Results

This study found that the SA group showed consistent cost savings compared to GA counterpart, with notable significance overall, OR supplies and in-patient costs. The SA group exhibited savings in total, OR supplies/medications, and inpatient costs, respectively. Within OR supplies/medications, savings were seen specifically within the sedative and anesthetic, intravenous solutions, and blood pressure management subgroups, respectively. This finding may be due to general anesthesia involving a more extensive list of administered medications than spinal anesthesia. Under general anesthesia, medications are needed to induce unconsciousness, maintain sedation, provide muscle relaxation, and often include a combination of sedatives, anesthetics, muscle relaxants, and analgesics including opioids [30]. In contrast, spinal anesthesia is a regional technique that usually requires only a single injection of a local anesthetic, occasionally with an opioid for extended pain relief [31]. Fewer medications are required overall, as patients remain awake and need fewer sedatives or muscle relaxants, making spinal anesthesia generally less medication-intensive than general anesthesia. In this study, although intrathecal opioids were not used, all SA patients received intravenous propofol and opioid sedation intraoperatively to maintain comfort throughout the procedure. The inability to convert to general anesthesia in the prone position underscores the importance of thoughtful case selection and close coordination between the surgical and anesthesia teams to ensure patient safety and procedural success. Additionally, current research has established general anesthesia, particularly opioids as a contributor to intraoperative hypotension [32,33]. This may explain the increased cost of intravenous solutions and blood pressure management observed in this study because of medication needed to induce and maintain general anesthesia in the GA group.

Additionally, SA was found to have significant cost savings with regard to inpatient costs, specifically in labs and blood tests, imaging and diagnostics, evaluations and therapies, hospital care and medications, respectively. This may be due to general anesthesia requiring closer monitoring and potential hospital stay [34]. This is plausible in the setting of this study’s finding of increased length of stay in the GA group. Additionally, anti-emetics and supportive medications are often required during and after surgery to manage potential side effects, such as nausea and vomiting [35]. Furthermore, awake spine surgery is linked to less postoperative nausea and vomiting when compared to GA [12] which may culminate in lower costs spent on medications to address such complications. It is also important to note that itemized cost data, including surgical tools, hemostatic agents, and implants, were retained in the analysis to reflect total inpatient cost from the hospital perspective. While implant costs, in particular, are unlikely to be influenced by anesthesia type, their inclusion does not imply a causal relationship, and they were not analyzed independently.

Looking at non-cost outcomes, LOS was significantly shorter in the SA group compared to the GA group, despite other postoperative outcomes showing no significant differences. This finding aligns with previous research demonstrating reduced LOS in awake spine surgery patients and is expected given the enhanced recovery typically associated with awake procedures [12]. However, given the small sample size and retrospective nature of the study, these findings should be interpreted as exploratory. While coarsened exact matching was used to minimize baseline differences, residual clinical or unmeasured confounding may still influence the observed outcomes. Further prospective studies are warranted to validate these results.

### 4.3. Comparison with Existing Literature

This study reinforces the existing literature that identifies SA as a cost-effective option in spine surgery, specifically one that reduces both intraoperative and postoperative costs without impacting quality of care. Prior studies have observed that SA is time- and cost-efficient with minimal impact on clinical and patient-reported outcomes [14,15,16,17,18,19,20,21,22,23,24,25,26]. Differences between this and other studies, especially in reported recovery metrics, likely stem from variations in institutional practices, patient selection, and surgical techniques. Broadly, however, this study’s findings are consistent with the previous literature on lumbar spine surgery, indicating that SA is a cost-effective alternative to GA in lumbar spine surgery while maintaining comparable clinical efficacy and patient outcome measures.

Of the two studies analyzing cost differences between SA and GA in TLIFs, both demonstrate that SA offers advantages over GA in terms of lower blood loss, reduced anesthesia and OR time, improved postoperative analgesia, and reduced anesthesia-related costs [16,17]. The Sarkar et al. study, performed in India, provides additional evidence regarding reduced nausea and vomiting in SA and identified a moderate (−10%) cost reduction [17]. Sekerak et al., performed in the United States, highlights that both SA and GA groups demonstrate significantly shorter operative and recovery times, reduced postoperative pain and opioid usage, and achieved slight cost savings of $812.31 (−5.6%) compared to GA [16]. Interestingly, both groups experienced similar rates of nausea, vomiting, and postoperative adverse events16. Sarkar et al. found that SA was associated with significantly reduced blood loss, shorter operating room time, better postoperative pain management, lower incidence of nausea and vomiting, and a reduction in costs compared to GA [17]. However, neither of these studies employed a matched cohort for comparisons. Together, these studies agree with these findings that SA could be a cost-effective and clinically beneficial alternative to GA in TLIFs, with fewer complications and improved patient outcomes. These findings add nuance to the current understanding of anesthesia choice in spine surgery, affirming SA’s role as a feasible and beneficial approach within appropriate clinical contexts.

Our study used a matched cohort, was performed in the US, and identified a larger amount of savings, $7787.16 (−24.51%), making it the first of its kind. By demonstrating a substantial cost reduction associated with SA selection in TLIF, our findings provide compelling evidence for the economic and clinical benefits of spinal anesthesia in this surgical context. This study’s addition to the body of literature on anesthesia selection in TLIF highlights a significant opportunity to refine perioperative care in spine surgery, particularly in settings focused on value-based healthcare delivery. These results underscore the potential for hospitals and surgical centers to optimize resource allocation while maintaining high-quality patient outcomes, reinforcing the growing movement toward cost-effective, patient-centered surgical strategies and delivery [20].

### 4.4. Clinical Relevance

With the increasing burden of DLSD, the economic benefits of SA in minimally invasive spine surgery underscore its role in promoting efficient resource use and supporting value-based care principles. Unlike GA, which necessitates extensive pharmacologic management and close postoperative monitoring, SA requires fewer anesthetics and sedatives, offering immediate cost savings and enhancing perioperative efficiency. This can facilitate faster patient mobilization and shorter recovery times—crucial in elective spine procedures where postoperative rehabilitation is essential. By reducing physiological disruption, SA not only aligns with modern healthcare goals of precision and economy but may also lessen the need for postoperative interventions, potentially improving patient satisfaction [16].

For spine surgeons, the implications of SA’s efficiency are compelling: it allows them to maintain operative control while potentially fostering smoother postoperative transitions that align clinical outcomes with patient-centered goals. In contrast, GA, though standard in spine surgery, imposes greater systemic demands due to its extensive pharmacologic load and requirement for intensive monitoring, which can lead to longer recovery times. These considerations suggest a more targeted anesthesia approach, particularly in TLIF surgeries, where SA can be optimally used for selected patients with stable cardiovascular and respiratory profiles, achieving a balance of risks and benefits [20,36]. Additionally, a length of stay less than 24 h allows due to spinal anesthesia allows patients to recover sooner and be discharged earlier. This swift discharge ensures that lumbar procedures can be safely conducted in outpatient clinics or ambulatory surgical centers, decreasing reliance on extended inpatient care and associated hospital-acquired complications [12,37]. This promotes increased patient health and satisfaction due to being able to recuperate in the more familiar and comfortable home environment, while simultaneously boosting the efficiency and accessibility of healthcare services.

In today’s healthcare landscape, where cost-effective, high-quality care is prioritized, SA stands out for its advantages in reducing hospital stay length, medication use, and potential postoperative complications. From a spine surgery perspective, SA’s shorter recovery trajectories may expedite postoperative mobilization and rehabilitation, aligning well with enhanced recovery after surgery (ERAS) protocols. For anesthesiologists, SA offers a modality that mitigates the metabolic and hemodynamic stress associated with GA, potentially reducing the overall anesthetic load and supporting smoother postoperative transitions. Additionally, the decreased need for opioids and adjunct medications with SA may further enhance recovery by reducing risks of adverse effects like nausea and prolonged sedation.

### 4.5. Future Research Directions

This study specifically examined direct hospital costs between two similar groups, assuming that a comparable cohort of patients undergoing the same procedure would have similar indirect/overhead costs. Given this, cost differences observed can be primarily attributed to intraoperative and postoperative hospital expenses rather than variations in indirect expenditures. Additionally, this analysis does not assess revenue or return on investment. However, it is reasonable to infer that, assuming a similar payer mix between the two groups, revenue would be similar as well. In such a case, a lower cost structure in the SA group would translate to a higher net margin. Since we are not examining revenue in this study and given the small sample size, no definitive conclusions can be drawn regarding overall financial impact beyond direct cost savings.

While these findings are encouraging, further research is warranted to assess SA’s long-term clinical and financial impact in spine surgery. Prospective studies with larger and more generalizable participant groups comparing SA and GA in TLIF would strengthen the evidence base, allowing for robust assessments of both immediate and lasting outcomes, including quality of life and rehabilitation metrics. Additionally, expanding investigations to include other spine surgery types and exploring SA’s impact on various patient demographics, particularly including elderly or high-risk patients, would provide valuable insights into its broader applicability.

Examining SA within the framework of ERAS protocols is another promising area. By integrating SA into ERAS pathways, further research can focus on potential reductions in recovery time, complications, and healthcare resource utilization. Research into SA’s influence on inflammatory responses, pain modulation, and hemodynamic stability during spine surgery could also elucidate its potential to optimize perioperative physiology, paving the way for more nuanced patient-specific anesthesia strategies.

Further work should also address the technical and physiological challenges of performing spinal anesthesia for lumbar fusion procedures. These include the potential for intraoperative patient movement, hemodynamic instability in the prone position, and the need for supplemental sedation or analgesia. Development and validation of standardized protocols—encompassing anesthetic dosing, sedation regimens, and intraoperative monitoring—will be essential for ensuring both patient safety and reproducibility across settings.

Lastly, while cost is an important factor in evaluating spinal anesthesia’s value, future research should continue prioritizing patient safety, feasibility, and long-term outcomes as primary endpoints.

### 4.6. Limitations

This study’s retrospective design presents inherent limitations, including potential selection bias if anesthesia choice was influenced by factors like surgeon preference or patient health status. Although there were no statistically significant differences between the two groups, the GA group trended to have higher incidence of co-morbid conditions (including hypertension, diabetes, smoking, and repeat spine surgery) and had a non-significant higher ASA status. In combination, this could reflect a more complex group of patients. Additional limitations include reliance on administrative and EMR data, which introduces challenges with coding accuracy and incomplete records, potentially affecting cost data precision. The generalizability of these findings may also vary across institution, patient demographics, and healthcare systems, as differences in protocols, healthcare models, and patient characteristics can influence cost-effectiveness and clinical outcomes. Additionally, limitations in capturing indirect and long-term costs, such as rehabilitation or follow-up care, could impact overall conclusions given that retrospective cost data often lack consistency, especially in indirect expenses. Potential sources of bias, including unmeasured confounders like surgeon experience, institutional protocols, or patient preferences, may further influence outcomes. Prospective trials with controlled data collection, bias-mitigation techniques like propensity score matching, and enhanced data capture methods such as sensitivity analyses are recommended to support more granular, reliable, and broadly applicable analyses. It is also important to emphasize that the feasibility and safety of spinal anesthesia in lumbar fusion are highly dependent on thoughtful case selection and close collaboration between surgical and anesthesia teams. The inability to convert to general anesthesia once a patient is in the prone position necessitates proactive intraoperative planning, advanced surgeon expertise, and limits generalizability to cases where stable anesthetic conditions can be anticipated. These technical considerations further underscore the need for prospective validation and the cautious interpretation of our early results.

## 5. Conclusions

In conclusion, this study positions spinal anesthesia as a promising, cost-effective option for TLIF surgery that offers substantial economic advantages without compromising patient care. The findings suggest that SA may optimize resource utilization and streamline perioperative care, aligning with the broader goals of cost-effective, high-quality spine surgery. However, these results should be interpreted with nuanced context due to limited generalizability. Additionally, cost alone should not drive anesthetic decision-making; patient safety, procedural feasibility, and the ability to mitigate risks associated with general anesthesia remain paramount. Future research with larger cohorts, prospective data, and longer-term follow-up—including functional and quality-of-life outcomes—will be crucial in affirming SA’s role and informing standardized protocols for its safe and effective use in spine surgery.

## Figures and Tables

**Table 1:** Depicting all major cost categories and their respective minor cost categories.

Major Category	Minor Category		
Anesthesia Time	Anesthesia Room Fee and Per Minute		
Implants	Cages	Caps	Grafts
	Rods	Screws	Spacers
	Tulips		
In-Patient Costs	Evaluations and Therapy	Imaging and Diagnostic Tests	Labs and Blood Tests
	Hospital Care	Medical Devices	Medications
	Treatments and Procedures		
OR Supplies	Analgesics	Anti-nausea Medications	Antibiotics
	Burrs and Drill Bits	Drapes	Guidewires and Stylets
	Hemostatic Agents	Imaging Costs	Intravenous Solutions
	Local Anesthetics	Needles and Cannulas	Other Medications
	Pressure Management	Probes and Stimulators	Sedatives and Anesthetics
	Steroids	Surgical Tools and Irrigation	
OR Time	OR Fee and Per Minute		
PACU Time	Time spent in PACU		

**Table 2:** Depicting patient demographics and characteristics.

	Spinal Anesthesia (n = 9)	General Anesthesia (n = 9)	*p*-Value
Age (years)	65.8 ± 6.8	67.2 ± 7.9	*p* = 0.37
Gender			*p* = 0.63
Male (%n)	5 (55.6%)	6 (66.7%)	
Female (%n)	4 (44.4%)	3 (33.3%)	
BMI (kg/m^2^)	27.7 ± 3.3	29.0 ± 5.1	*p* = 0.36
History of Smoking	3 (33.3%)	4 (44.4%)	*p* = 0.58
History of Hypertension	5 (55.6%)	9 (100.0%)	*p* = 0.09
History of Diabetes	3 (33.3%)	5 (55.6%)	*p* = 0.46
History of Heart Disease	3 (33.3%)	6 (66.7%)	*p* = 0.25
History of Pulmonary Disease	5 (55.6%)	5 (55.6%)	*p* = 1.00
History of Renal Disease	7 (77.8%)	4 (44.4%)	*p* = 0.414
History of Past Spine Surgery	2 (22.2%)	4 (44.4%)	*p* = 0.45
Level of Surgery			*p* = 1.00
L3/L4	1 (11.1%)	1 (11.1%)	
L4/L5	7 (77.8%)	7 (77.8%)	
L4-S1	1 (11.1%)	1 (11.1%)	
Type of Surgery			*p* = 1.00
MIS-TLIF	5 (55.6%)	5 (55.6%)	
Perc-LIF	4 (44.4%)	4 (44.4%)	
ASA Grade			*p* = 0.11
2	5 (55.6%)	1 (11.1)	
3	4 (44.4%)	7 (77.8%)	
4	0 (0.00%)	1 (11.1%)	

**Table 3:** Depicting intraoperative and postoperative surgical and patient centered outcomes.

	Spinal Anesthesia (n = 9)	General Anesthesia (n = 9)	*p*-Value
Estimated Blood Loss (mL)	50.0 ± 21.7	81.3 ± 75.3	*p* = 0.50
OR time (min)	181.0 ± 61.4	213.0 ± 90.3	*p* = 0.50
Length of stay (hours)	12 ± 11.1	78 ± 42.1	*p* = 0.04
Post-Op Opioid Use (mg)	20.1 ± 7.6	45.6 ± 11.8	*p* = 0.13
30-day readmission			*p* = 0.30
No Readmission (%n)	9 (100.0%)	8 (88.9%)	
Readmission (%n)	0 (0.0%)	1 (11.1%)	
Difference in NDI	0.9 ± 3.5	0.18 ± 0.5	*p* = 0.60
Difference in ODI	−12.7 ± 42.1	−20.0 ± 24.7	*p* = 0.62
Difference in VAS B	−3.0 ± 4.8	−3.8 ± 3.2	*p* = 0.59
Difference in VAS L	−1.9 ± 5.4	−3.2 ± 3.7	*p* = 0.49
Difference in PROMIS Pain	1.4 ± 18.5	−3.7 ± 32.7	*p* = 0.75
Difference in PROMIS PF	−7.7 ± 40.5	−4.6 ± 47.9	*p* = 0.99
Difference in PROMIS GH Physical	−4.7 ± 29.7	−7.8 ± 21.3	*p* = 0.82
Difference in PROMIS GH Mental	−5.7 ± 34.0	−10.7 ± 27.4	*p* = 0.75

## Data Availability

The data presented in this study are available on request from the corresponding author.
